# The identification of candidate effective combination regimens for pancreatic cancer using the histoculture drug response assay

**DOI:** 10.1038/s41598-020-68703-x

**Published:** 2020-07-20

**Authors:** Eunsung Jun, Yejong Park, Woohyung Lee, Jaewoo Kwon, Song Lee, Moon Bo Kim, Ji Sun Lee, Ki Byung Song, Dae Wook Hwang, Jae Hoon Lee, Robert M. Hoffman, Song Cheol Kim

**Affiliations:** 1 Division of Hepato-Biliary and Pancreatic Surgery, Department of Surgery, Asan Medical Center, AMIST, University of Ulsan College of Medicine, 388-1 Pungnap-2 Dong, Songpa-gu, Seoul, 05505 South Korea; 2 Department of Convergence Medicine, Asan Institute for Life Sciences, Asan Medical Center, University of Ulsan College of Medicine, Seoul, 05505 Korea; 3 Asan Institute for Life Sciences, Asan Medical Center, University of Ulsan College of Medicine, Songpa-gu, Seoul, 05505 Korea; 4 MetaBio, Inc., Gangdong-gu, Seoul, 05327 Korea; 5 Department of Surgery, University of California, San Diego 92103-8220, CA 92111 USA; 6 AntiCancer, Inc., 7917 Ostrow Street, San Diego, CA 92111 USA

**Keywords:** Biological techniques, Cancer, Drug discovery

## Abstract

The prognosis for patients with pancreatic cancer is extremely poor, as they are resistant to first line chemotherapy. The long-term goal of this study was to identify effective combination chemotherapy for pancreatic cancer using pancreatic cancer surgical specimens in the histoculture drug response assay (HDRA) based on three-dimensional culture of tumour fragments, which maintains nature tumour histology in vitro. From 2015 to 2017, the HDRA was performed with tumour specimens from 52 pancreatic cancer patients from Asan Medical Hospital. First, combination drug regimens showed higher drug efficacy and less patient variation than single drugs. Initially, 5-Fluorouracil(5-FU)/Belotecan/Oxaliplatinum and Tegafur/Gimeracil (TS-1)/Oxaliplatinum/Irinotecan were found to be effective. Second, we were able to correlate the efficacy of some drugs with tumour stage. Third, when designing new combination regimens containing 5-FU or gemcitabine, we could identify more effective drug combinations. This is the first study to demonstrate usefulness of the HDRA for pancreatic cancer. Using this technique, we could identify novel candidate combination drug regimens that should be effective in treating pancreatic cancer.

## Introduction

The prognosis of patients with pancreatic cancer is poor; the 5-year survival rate is less than 10%^1,2^, and it is predicted to be the second most common cause of cancer-related deaths by 2030^3^. In particular, pancreatic cancers is characterized by rapid tumour growth, frequent recurrence, and early metastasis, which require intensive adjuvant chemotherapy^4,5^. During the last decades, adjuvant chemotherapy has been developed for pancreatic cancer. As a representative example, the survival rate of patients receiving mFOLFIRINOX has been reported to be more than doubled in selected cases^6,7^. In addition, numerous clinical trials are underway with various combination regimens based on 5-FU, gemcitabine, and nab-paclitaxel^6,8^. However, pancreatic cancer is recalcitrant to first-line chemotherapy. Due to the aggressive progression of the disease, rapid selection of the most efficacious chemotherapy for each patient could improve patient outcome^9,10^. Numerous clinical studies have been performed on pancreatic cancer using a combination of radiation therapy, conventional chemotherapy, or various immunotherapies^11–13^. In addition, clinical studies on targeted and personalized therapy based on tumour biomarkers such as CCR2, PARP, PDH, STAT3, and mesothelin are also ongoing^14,15^. Especially, attempts to overcome the extensive extracellular matrices of pancreatic cancer have been made^16,17^. However, unlike lymphoma, breast cancer, and lung cancer, whose survival rate has improved through the discovery of novel treatment, pancreatic cancer remains recalcitrant^18–20^.

The histoculture drug response assay (HDRA) is based on three dimensional culture of tumour fragments that maintain the original cancer–stromal histological architecture^21,22^. The HDRA can identify effective chemotherapy drugs based on inhibition of tumour viability. Unlike other in vitro assays, the HDRA can maintain the tumour original microenvironment. Therefore, the HDRA correlated with patient survival in many clinical trials and may be more useful for cancers that have an extensive stromal component in the tumour microenvironment. A common characteristic of pancreatic cancer is desmoplasia, which involves the deposition of fibroblasts and extracellular matrix components into the tumour microenvironment^23^. Since the desmoplastic stroma acts as a physiological barrier for anticancer drugs, it is essential to consider the desmoplasia of pancreatic cancer in order to improve chemotherapy efficacy^16,24^.

In the present study, we used the HDRA to identify candidate effective chemotherapy drug combinations using pancreatic cancer surgical specimens. This is the first HDRA study for pancreatic cancer.

## Results

### Patient characteristics

Of the 52 patients enrolled in this study, 32 were male (61.5%) and the average age was 63.7 years (Table 1). The average values of preoperative blood tests were WBC 6.2 (× 10^3^/µl), Hb 12.77 (g/dl), albumin 3.69 (g/dl), and CA 19–9 228.1 (U/ml). Only 5 patients (9.6%) received chemotherapy before surgery. Distal pancreatectomy was the most common surgical technique (57.7%), and the average tumour size measured after surgery was 3.72 cm. Forty-nine cases (94.2%) were diagnosed as ductal adenocarcinoma, and advanced stages (T2, N1/2) were more frequent than early stages (T1, N0).

**Table 1:** Host and tumor factors of enrolled patients.

Factors	Values
Sex (female/male), N (%)	20/32 (38.5%/61.5%)
Age, years, Avg ± SD (range)	63.69 ± 7.93 (46–79)
BMI (kg/m^2^), Avg ± SD (range)	24.3 ± 3.56 (17.63–35.13)
**Preoperative laboratory, Avg ± SD (range)**	
WBC (× 10^3^/µl)	6.2 ± 1.79 (3.8–12.2)
Hb (g/dl)	12.77 ± 1.68 (8.9–16.2)
Albumin (g/dl)	3.69 ± 0.36 (2.8–4.3)
Total bilirubin (mg/dl)	1.01 ± 2.29 (0.1–13.6)
CA 19-9 (U/ml)	228.1 ± 436.9 (0.6–2003.0)
Preoperative chemotherapy (yes/no), N (%)	5/47 (9.6%/90.4%)
Operation (PPPD/DPS/TPS), N (%)	20/30/2 (38.5%/57.7%/3.8%)
Tumor size (cm), Avg ± SD (range)	3.72 ± 2.22 (0.3–14.8)
Tumor_differentiation (wel/mod/por), N (%)	1/41/7 (2.0%/83.7%/14.3%)
Lymphovascular invasion (absent/present), N (%)	15/37 (28.8%/71.2%)
Perineural invasion (absent/present), N (%)	11/41 (21.2%/78.8%)
T stage (T1/T2/T3), N (%)	9/29/14 (17.3%/55.8%/26.9%)
N stage (N0/N1/N2), N (%)	24/17/11 (46.2%/32.7%/21.2%)
**Pathologic diagnosis, N (%)**	
Ductal adenocarcinoma	49 (94.23%)
Adenosquamous carcinoma	1 (1.92%)
Neuroendocrine carcinoma	1 (1.92%)
Hepatoid adenocarcinoma	1 (1.92%)

*BMI* body mass index, *WBC* white blood cell, *Hb* hemoglobin, *PPPD* pylorus preserving pancreaticoduodenectomy, *DPS* distal pancreatectomy with splenectomy, *TPS* total pancreatectomy with splenectomy.

### Inhibitory rate of drugs in the HDRA

**Figure 1:**
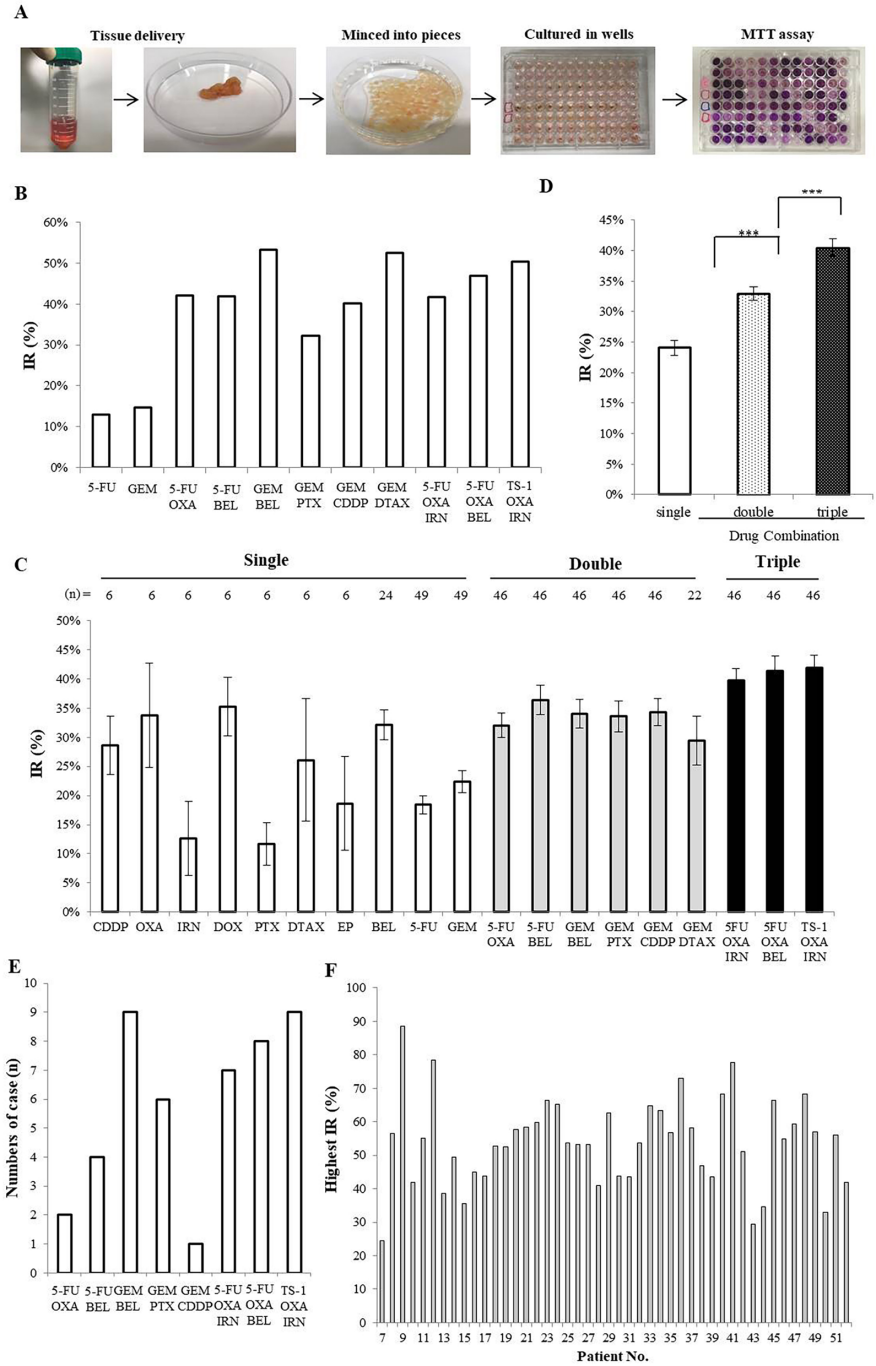
Comparison of inhibitory rates (IRs) of single and combination drug regimens. **(A)** The patient's tissue was excised, divided into fragments, and placed in culture medium, treated with drugs and tested with the MTT assay. Cell viability with various drugs was determined in the HDRA. **(B)** Example HDRA results from patient 27. **(C)** Comparison of IR average values for each drug regimen. **(D)** Comparison of IRs between single drug and combination regimens (Kruskal–Wallis test, *** p < 0.001). **(E)** The number of drug combinations with the highest IR among the cases tested in the HDRA. Compared to other regimens, GEM/DTAX was excluded due to the small number of assays. **(F)** Summary of the highest IR values for each patient. Results from patients 7 to 52 with combination regimens.

The inhibitory rate (IR) of various drug regimens in the HDRA was determined using the MTT (3-(4,5-dimethylthiazol-2-yl)-2,5-diphenyltetrazolium bromide) assay. As shown in Fig. 1A, the tissue obtained through surgery was transferred to the laboratory, divided into small tumor pieces, and the pieces were placed in each well of 96 well plates. Phosphate buffered saline (PBS) was used in the control well, and drugs were added to the other wells. The plates were incubated for 72 h with the drugs and the inhibitory rate of each drug was determined with the MTT assay (Fig S1, Table S1). In the first six patients, we analyzed the IRs of nine single drugs (Table 2). Among the single drugs, OXA, DOX, and BEL showed superior inhibitory rates compared to the other drugs tested. With this initial experiment, we determined that it is possible to distinguish the efficacy drugs for pancreatic cancer in the HDRA. Subsequently, 46 patients were tested in the HDRA, using single clinically-approved drugs and combination regimens. As a representative example, the IR values for patient No. 27 are shown in Fig. 1B. The IRs for each drug or combinations demonstrated that the efficacy of the drug regimen improved in the order of a single drug (24.0%); a two-drug combination (33.0%); a three-drug combination (40.5%). The differences between IR values were relatively small between various triple-drug combination regimens, even though they comprised different drugs (Fig. 1C, D). The efficacy of these regimens did not differ significantly whether pre-operative chemotherapy was previously administered to the patient or not (Fig. S2). GEM/BEL in the two drug combination and TS-1/OXA/IRN in the three-drug combination were the most effective regimens (Fig. 1E). The highest IR values for each patient among 46 patients using a combination regimen are shown in Fig. 1F. The minimum and maximum values of the highest IR in the HDRA were 24.5% and 88.6%, respectively, for the present cohort of patients.

**Table 2:** Inhibitory rate in the histoculture drug response assay (HDRA).

No	Inhibitory rate (%) of drugs																			
	Single regimens										Combination regimens									Avg
	CD DP	OXA	IRN	DOX	PTX	DT AX	EP	BEL	5FU	GEM	FOx	FOxIri	FBe	FBeOx	GBe	GT	GC	GD	TsOxIri	
											5FU OXA	5FU OXA IRN	5FU BEL	5FU BEL OXA	GEM BEL	GEM PTX	GEM CDDP	GEM DTAX	TS-1 OXA IRN	
1	27.8	**46.9**	10.8	37.2	22.9	22.9	7.4		16.0	29.1										24.6
2	10.2	**65.5**	10.4	41.8	4.3	74.6	4.5		4.5	52.1										29.8
3	21.5	41.3	2.8	**44.4**	8.9	3.0	7.0		10.7	5.3										16.1
4	**45.3**	19.9	43.5	22.4	6.1	4.9	42.1		28.4	12.6										25.0
5	**28.2**	3.3	2.4	18.2	5.0	25.3	4.8		3.1	4.9										10.6
6	38.7	26.2	5.7	**47.7**	23.2	25.9	46.1		18.3	27.5										28.8
7											16.4	9.6	3.4	10.5	**24.5**	18.4	5.9	5.5	8.9	11.5
8											**56.5**	53.6	20.0	46.3	12.3	46.5	17.8	19.8	49.2	35.8
9									21.2	18.3	55.1	51.0	48.7	**88.6**	22.3	45.7	50.7	45.0	45.2	44.7
10											24.9	35.2	16.8	24.3	28.6	33.0	27.3	**41.8**	22.0	28.2
11									8.7	22.9	26.3	37.0	40.0	**43.3**	26.4	55.0	22.5	17.2	19.5	29.0
12									21.0	55.8	55.5	55.5	30.7	78.4	**67.4**	**67.4**	65.5	72.2	60.5	57.3
13									12.9	16.4	20.3	20.0	22.1	**38.7**	8.8	21.3	24.1	16.4	15.5	19.7
14									26.1	37.1	27.9	27.5	**49.5**	46.9	31.1	40.5	42.9	41.0	40.1	37.3
15									9.7	6.5	12.4	15.8	7.3	26.9	28.7	**35.6**	31.2	7.4	6.9	17.1
16									14.3	21.3	17.4	36.8	16.5	31.9	16.8	22.7	43.4	24.9	**44.9**	26.4
17									4.1	4.6	37.1	41.3	4.2	15.9	17.0	4.9	29.7	29.8	**43.9**	21.1
18									18.4	12.5	20.9	20.3	28.3	52.7	33.3	11.7	41.6	4.8	**48.5**	26.6
19									40.4	35.6	10.8	45.0	42.3	52.6	18.0	24.9	30.0	8.6	**52.4**	32.8
20									15.1	18.3	40.9	47.7	25.9	43.4	18.6	36.7	46.4	20.8	**57.7**	33.8
21									5.8	35.1	14.0	47.3	52.4	56.0	46.5	26.3	38.4	22.5	**58.4**	36.6
22									40.6	17.8	49.3	51.7	59.5	44.9	35.6	24.3	21.7	13.0	**59.8**	38.0
23									4.6	40.8	37.4	38.4	64.1	9.2	65.3	60.8	64.0	61.0	**66.4**	46.6
24									25.3	32.2	35.1	53.4	55.6	59.0	**65.3**	28.6	59.5	50.8	60.1	47.7
25									7.8	9.1	19.7	**53.6**	47.9	14.7	26.9	11.7	17.8	17.6	49.1	25.1
26									11.4	19.7	10.6	6.4	3.5	11.4	42.9	20.6	30.1	**53.3**	26.2	21.5
27									12.9	14.7	42.2	41.7	41.9	47.0	**53.3**	32.3	40.1	52.5	50.4	39.0
28									10.9	8.9	31.1	**41.0**	23.5	19.8	8.3	16.0	12.7	21.2	31.8	20.5
29								28.3	7.2	20.0	30.2	32.3	44.6	49.3	23.7	**62.6**	35.4	–	43.5	34.3
30								15.2	8.2	9.7	30.4	37.5	21.2	34.7	8.5	29.7	27.9	–	**43.7**	24.2
31								13.4	11.6	11.8	20.0	43.1	24.6	24.7	15.9	15.3	11.1	–	**43.6**	21.4
32								46.0	7.7	9.7	35.2	50.4	45.8	39.6	37.8	**53.6**	25.4	–	20.1	33.8
33								45.6	15.1	12.3	16.8	24.0	44.7	44.9	42.4	58.7	**64.7**	–	41.2	37.3
34								18.2	18.7	43.0	31.7	46.9	46.4	48.7	**63.3**	59.5	40.7	–	43.3	41.9
35								38.3	22.7	14.9	41.1	**56.8**	24.6	27.5	33.3	55.6	25.1	–	51.0	35.6
36								20.5	25.6	13.4	34.9	39.3	46.9	54.7	50.4	**73.0**	58.1	–	39.9	41.5
37								15.0	9.9	20.6	11.4	33.7	24.8	32.6	28.7	**58.1**	32.3	–	35.1	27.5
38								46.1	21.4	33.1	26.5	27.6	36.3	22.9	**46.9**	31.3	40.4	–	28.3	32.8
39								37.0	18.3	11.2	23.1	41.0	31.2	40.5	**43.6**	21.5	10.8	–	38.4	28.8
40								34.9	35.5	52.9	60.5	**68.2**	50.3	65.5	34.3	43.5	41.3		62.3	49.9
41								40.5	40.4	38.1	48.8	52.0	**77.7**	52.1	55.5	40.5	53.0		60.8	50.9
42								38.8	19.8	30.6	46.5	42.7	**51.0**	43.3	48.0	12.5	36.0		43.3	37.5
43								10.6	16.0	7.9	20.9	**29.4**	25.5	28.3	13.4	11.9	13.2		27.0	18.6
44								28.7	13.3	28.3	28.8	**34.6**	25.8	28.4	33.9	11.0	32.3		31.9	27.0
45								41.2	40.9	16.7	46.9	66.2	61.6	**66.5**	35.3	46.8	33.9		53.2	46.3
46								53.2	27.8	24.3	37.2	46.6	29.3	**54.8**	39.1	30.8	40.9		41.0	38.6
47								36.4	11.7	15.1	**59.3**	39.6	50.4	55.9	58.1	22.0	51.1		55.3	41.4
48								18.6	43.7	22.4	52.9	60.2	46.2	60.2	5.9	45.4	45.7		**68.4**	42.7
49								44.4	30.1	31.7	39.5	34.3	41.1	42.7	57.1	32.4	9.8		**42.9**	36.9
50								22.0	10.0	16.0	9.0	21.0	28.0	**33.0**	19.0	10.0	13.0		17.0	18.0
51								44.0	41.0	16.0	41.0	42.0	**56.0**	46.0	33.0	17.0	45.0		41.0	38.4
52								35.0	13.0	**38.0**	21.0	28.0	35.0	42.0	42.0	19.0	31.0		36.0	30.9
Avg	28.6	33.8	12.6	35.3	11.7	26.1	18.6	32.2	18.4	22.4	32.1	39.7	36.4	41.3	34.1	33.6	34.4	29.4	41.9	32.1

The highest inhibitory rate was indicated in bold for each patient.

*CDDP* cisplatin, *OXA* oxaliplatin, *IRN* irinotecan, *DOX* Doxorubicin, *PTX* paclitaxel, *DTAX* docetaxel, *EP* epirubicin, *BEL* belotecan, *5-FU* 5-fluorouracil, *GEM* gemcitabine, *TS-1* tegafur, gimeracil.

### Comparison of IR with tumour stage or CA 19-9 vales

**Figure 2:**
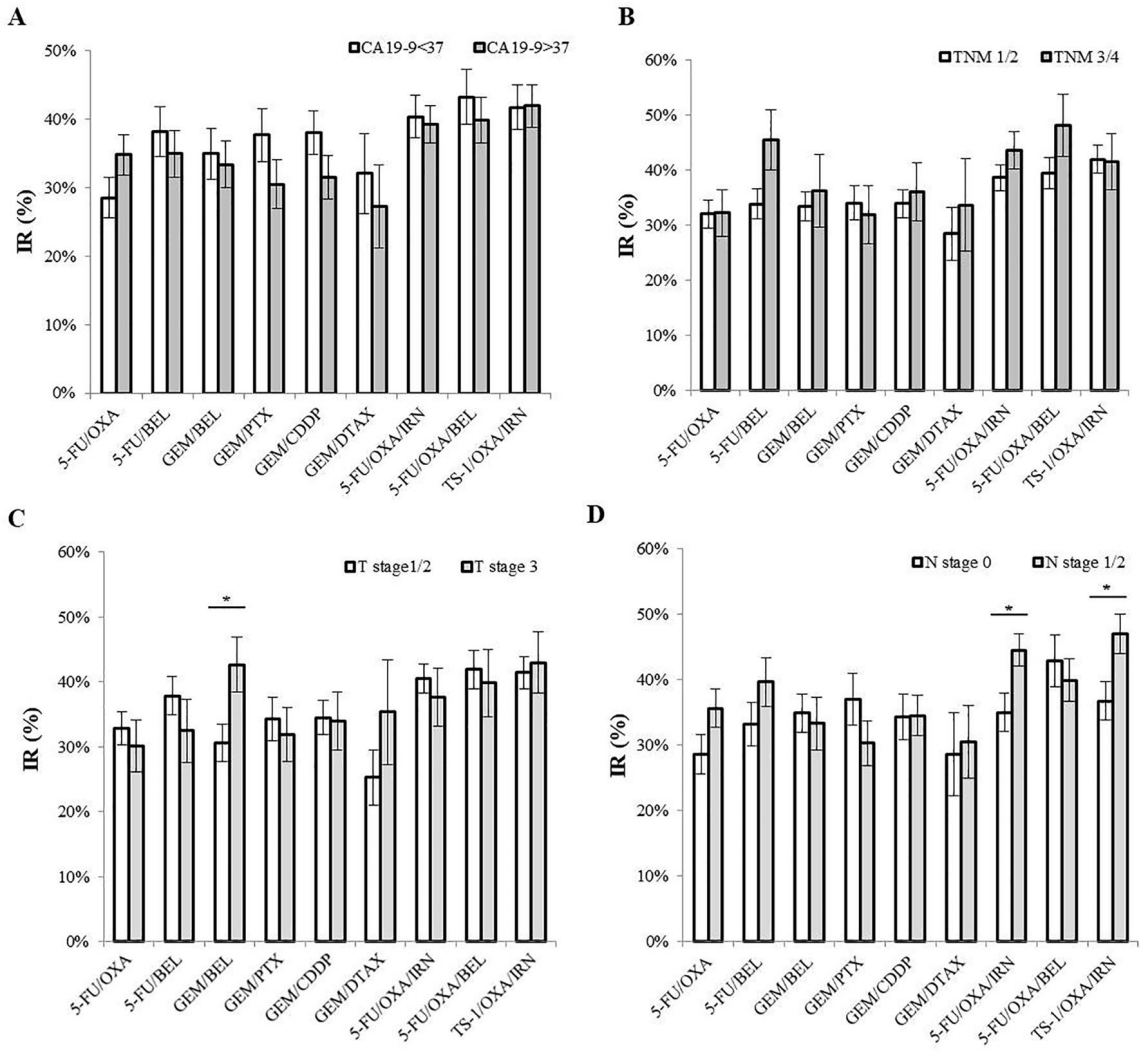
IR according to **(A)** concentration of CA19-9, **(B)** TNM stage, **(C)** T stage, and **(D)** N stage (n = 46; Kruskal–Wallis test, * p < 0.05).

**Figure 3:**
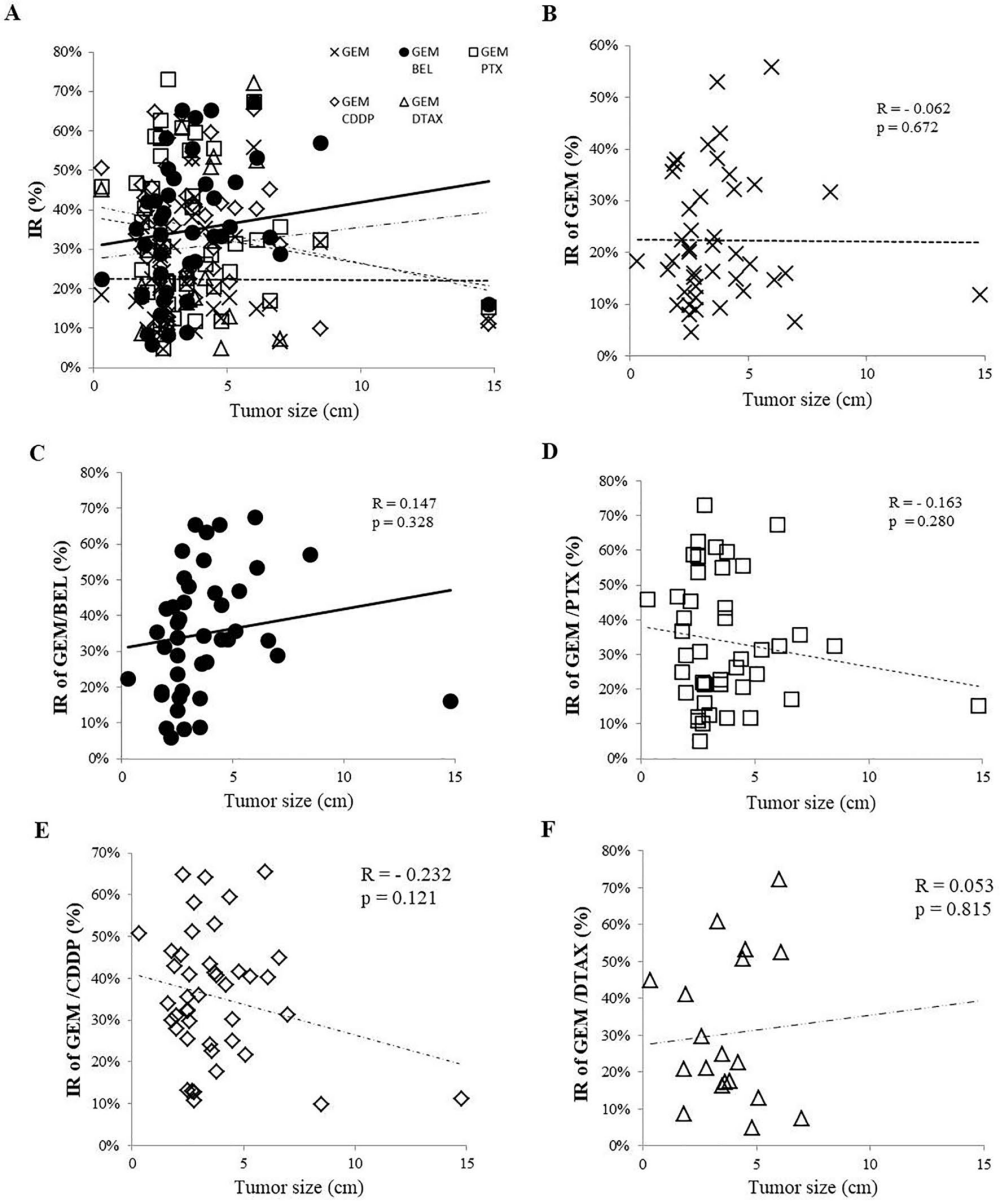
IRs of GEM, alone, and GEM-based combination regimens according to tumour size. **(A)** IRs of 5 regimens. **(B)** IR of GEM. **(C)** IR of GEM/BEL. **(D)** IR of GEM/PTX. **(E)** IR of GEM/CDDP. **(F)** IR of GEM/DTAX [n = 43 (exception, GEM/DTAX n = 19); Pearson correlation analysis].

We have observed that when CA19-9 values are 37 or higher, the IR of the drugs tends to be relatively low, and when the TNM stage is increased, the IR tends to increase, but there is no statistically-significant difference for these comparisons (Fig. 2A, B). However, when the T-stage and the N-stage were analyzed separately, there were significant differences. GEM/BEL was more effective at T-stage 3 and 5-FU/OXA/IRN and TS-1/OXA/IRN were more effective at N-stage 1/2. GEM/BEL and GEM/DTAX showed positive correlation with increasing tumour size, and GEM/PTX and GEM/CDDP showed negative correlation. GEM alone did not correlate with tumour stage (Fig. 3).

### IR of drug regimens based on 5-FU and GEM

**Figure 4:**
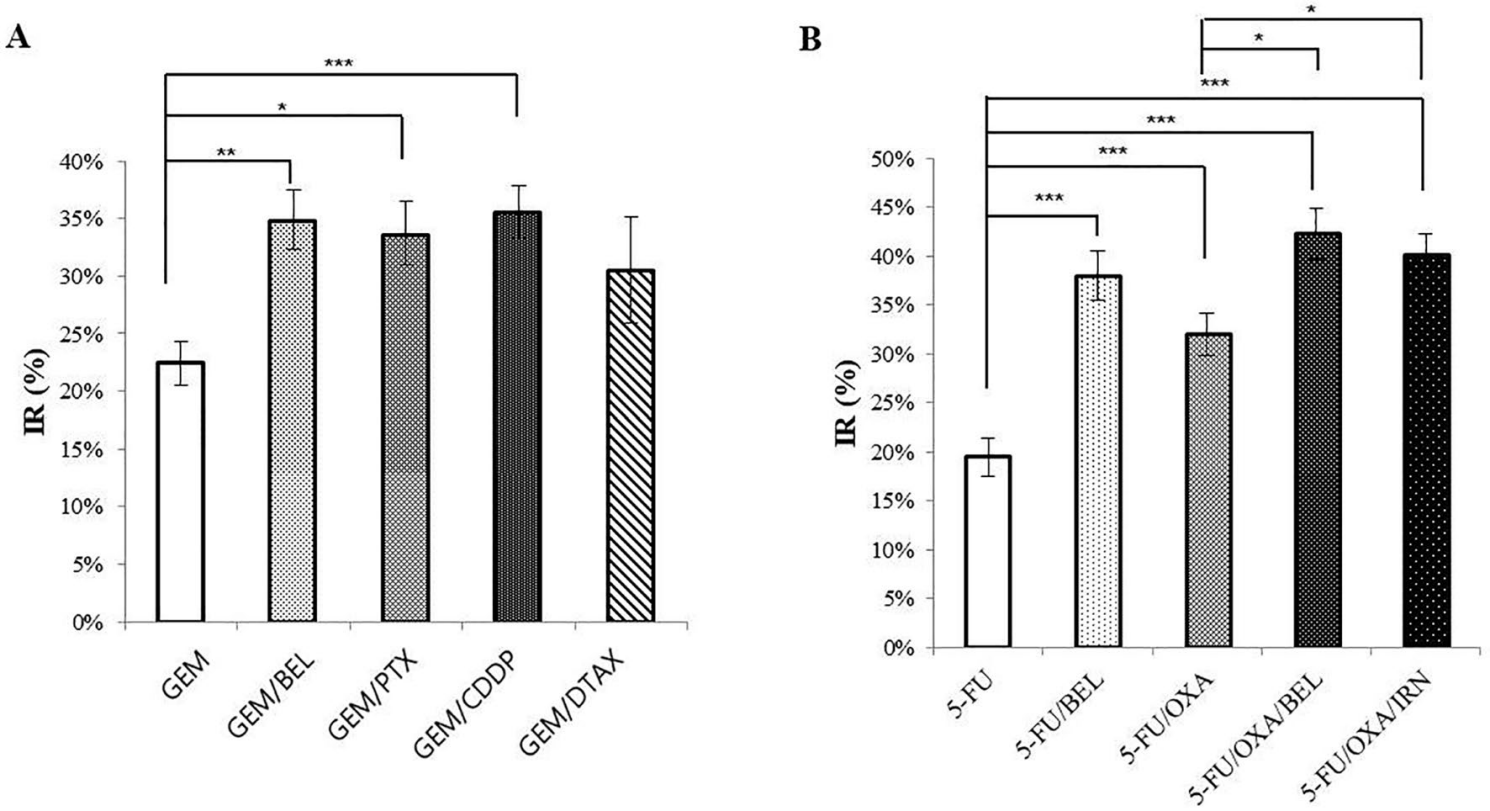
IRs of various combination drug regimens based on GEM and 5-FU. **(A)** Comparison of IR among GEM-based combination regimens [n = 43 (exception, GEM/DTAX n = 19); Kruskal–Wallis test,* p < 0.05, ** p < 0.005, *** p < 0.001]. **(B)** Comparison of IRs among 5-FU-based combination regimens (n = 43; Kruskal–Wallis test,* p < 0.05, ** p < 0.005, *** p < 0.001).

We compared combination regimens containing 5-FU or GEM, which are first-line chemotherapy in pancreatic cancer patients (Fig. 4). When combined with GEM, the efficacy of PTX, CDDP, and BEL was significantly increased. The IR of DTAX, when combined with GEM, was also increased, but the increase was not statistically significant. Next, 5-FU combined with BEL (p < 0.001) was more effective than combined with OXA (p < 0.005), and combining 5-FU/OXA and BEL (p < 0.05) was more effective than combining with IRN. In addition, the combination of 5-FU and BEL showed better efficacy than the combination of GEM with BEL (Fig. S3).

## Discussion

The HDRA was developed in 1987 using sponge gel-supported three-dimensional histoculture of tumour fragments from cancer patients^21^, which has many advantages including culture of intact tumour fragments which maintain native cancer-cell stroma-cell interaction allowing an in vivo-like environment much superior to 2D culture^25^. These 3D tumour fragments are also more realistic than “organoids” which are grown from “stem cells” which are not true tumour fragments and require long time periods to establish and do not contain stromal cells^26,27^. Patient-derived orthotopic xenografts that take 2–4 months to obtain drug response data, are therefore useful to select second-line and higher therapy^28^. The HDRA yield results in 3-days with an over 90% success rate and has been validated by numerous clinical trials^22,29–40^. For example, drug response in the HDRA was correlated to recurrence-free survival for stomach and colon cancer^22,31,33^. The responses of breast cancer to several drugs in the HDRA were correlated to recurrence-free survival^34,35^. Lung cancer response to nine drugs in the HDRA was correlated to the presence or absence of smoking history^36,37^. The HDRA has been used as a platform for the validation of drugs developed for cholangio-carcinoma^38^. Also, drug response in the HDRA of head and neck cancer was correlated to patient survival^29,39^.

Drug combinations that are efficacious in other tumour types were investigated in the present study for their efficacy on pancreatic cancer in the HDRA^41,42^. Given the enormous cost and R&D time required to develop an effective drug, it is necessary to interrogate existing drugs and their combinations as therapy for refractory tumours such as pancreatic cancer.

We used HDRA to rapidly determine the response of existing drugs and their combinations, and to show novel combination therapy, that included first-line pancreatic cancer drugs was more effective than monotherapy for pancreatic cancer. In the early phase in our experiments, we used nine single drugs that have been used in many cancers for a long time. Through this, it was confirmed that the HDRA assay can be useful for in pancreatic cancer. We tested several combination drug regimens based on 5-FU, GEM, and TS-1. The concentration of each drug was based on published HDRA papers with various tumour types^22,29–39^. The concentrations were adjusted in consideration of the characteristics of pancreatic cancer. We then verified the efficacy of the combination regimens (5-FU/OXA, 5-FU/OXA/IRN, GEM/PTX) currently used in patients with pancreatic cancer and then identified other new effective combinations. BEL- or TS-1-based combination regimens that are actively used on other cancers than pancreatic cancer. The HDRA experiments in the present study showed these and other novel combination regimen were effective in pancreatic cancer.

The HDRAs may be particularly useful for pancreatic cancer, which has a large amount of extracellular matrix (ECM) components that have a significant effect on the resistance to anticancer drugs. The HDRA is advantageous because the response to an anticancer drug can be obtained intuitively and quickly since the cancer and the various stromal cells, and extracellular matrix components in the tumour are not disrupted.

The present study identified three interesting points; First, combination regimens showed higher drug efficacy and less variation than a single drug. Drug combinations are likely more effective due to the complexity and heterogeneity of the tumour microenvironment^43,44^. Of the initial drug combinations we tested, 5-FU/BEL/OXA and TS-1/OXA/IRN regimens were the most effective. However, these have rarely been used to treat pancreatic cancer, but since they are specific for different targets, they will likely be effective in new drug combinations for this disease (Table S2). Based on additional research demonstrating why these combinations are more effective, new clinical trials may be justified.

Second, we correlated the efficacy of some drug combinations to the degree of tumour progression. As a tumour progresses, its microenvironment changes, and quantitative and qualitative changes in the drug target occur^45,46^. Therefore, the same drug can have varying efficacy, depending on the patients. Our results showed that GEM/BEL, 5-FU/OXA/IRN, and TS-1/OXA/IRN were more effective against advanced tumours. This may be due to changes in the tumour microenvironment or target genes or ability of the drug to reach into the tumour. The goal is to enable the selection of more effective drugs, especially combinations in the HDRA.

Third, better combination regimens containing 5-FU or GEM were designed. Even though there are many drugs available to patients, there is a lack of evidence on which combination of drugs will ultimately be more effective for each patient. Usually, drug combinations are designed based on the results of large clinical trials, but this strategy is very costly and time-consuming and does not consider the individuality of the patient. In our results, GEM/CDDP and 5-FU/BEL were found to be very effective combinations. Effective drug combinations in the HDRA can be rapidly identified, shorten the time to the next clinical trial and increase the success rate of the clinical trial.

Although the HDRA requires a relatively large amount of tumour tissue to evaluate drug efficacy, it can be overcome by obtaining sufficient tumour specimens after surgery. Especially, the HDRA can be a useful platform to rapidly identify drug responsiveness for each patient. Future clinical trials will determine the clinical efficacy of the novel drug combinations identified in the HDRA of pancreatic cancer in the present study.

We investigated the utility of the HDRA as a platform for identifying candidate effective drug regimens, especially combination therapy in pancreatic cancer. HDRA allowed us to identify the most effective candidate drug regimen for each patient and provided information that guided the selection of effective drug combination regimens. Further research is needed to design drug combinations that are optimal for each pancreatic cancer patient.

## Materials and methods

All experiments and methods were performed in accordance with relevant guidelines and regulations.

### Study design

Fifty-two patients who underwent surgery for pancreatic cancer at the Asan Medical Center between 2015 and 2017 were included in the study. Surgical specimens were obtained from the patents after informed consent with approval by the Institutional Review Board (IRB) of Asan Medical Center (IRB No. 2015-0480). Surgical specimens were transported in Hanks' balanced salt solution (HBSS; Gibco) to the MetaBio Laboratory, MetaBio Co., Inc., where the assay was conducted.

### Drug concentration for HDRA

Information about the drugs used in the study are summarized based on the KEGG (Kyoto Encyclopedia of Genes and Genomes) Drug Database (https://www.genome.jp/kegg/drug/, Table S2). Each anticancer drug used was purchased from a pharmaceutical company. Each drug was dissolved in 0.5% dimethyl sulfoxide (DMSO) or phosphate-buffered saline (PBS). The concentrations for each drug used in the study were as follows: 5-fluorouracil: 50 ug/ml; gemcitabine: 50 μg/ml; cisplatinum: 5 μg/ml; oxaliplatinum: 20 μg/ml; irinotecan: 40 μg/ml; doxorubicin: 6 μg/ml; paclitaxel: 5 μg/ml; docetaxel: 75 μg/ml; epirubicin: 10 μg/ml; belotecan: 20 μg/ml; and TS-1(tegafur/gimeracil): 500 μg/ml. Drug concentrations were calculated from pharmacodynamic parameters and clinical doses in the published papers and also modified from growth-inhibition studies using pancreatic cancer cell lines^22,33,47–49^.

### HDRA with the MTT end point

The method of Hoffman and colleagues was utilized employing the MTT end point, which has been validated in clinical trials^22,29,37^. Collagen sponge gels manufactured from pig skin were purchased from Health Design Inc. (Rochester, NY)^21,22^. The cancerous portion of the specimens was scissors-minced into 12 pieces approximately 1 mm in diameter, which were then randomly placed on each piece of the prepared collagen sponge gel in 12 wells of a 96-well plate. The plates were incubated for 3 days at 37℃ with the 11 drugs and PBS as control) dissolved in RPMI 1,640 medium containing 20% fetal calf serum in a humidified atmosphere containing 95% air and 5% CO_2_. After histoculture, 900 μL of Hank's Balanced Salt Solution containing 0.1 mg/μL collagenase (type I; Sigma, US) and 100 μL MTT (Sigma, US) solution were dissolved in 4 mg/μL PBS, and added to each culture well and incubated for another 2 h. After extraction with DMSO, the absorbance of the solution in each well was read at 540 nm. The absorbance/g of the histocultured tumour tissue was calculated from the mean absorbance generated by each culture well, and the tumour-tissue weight was determined prior to culture. The inhibition rate was calculated using the following formula:

$${\it{Inhibition \, rate }}( \% ) \, = \, ( {{1 } - {\it{ mean \, absorbance \, of \, treated \, tumour}}/{\it{g}}/{\it{mean \, absorbance \, of \, control \, tumour}}/{\it{g}}} ) \, \times { 1}00.$$

### Clinical data collection of patients

Medical records for each patient were retrospectively reviewed for surgery-, oncology-, and survival-related data. Age, sex, and BMI (kg/m^2^) were confirmed. Preoperative blood tests included WBC, Hb, albumin, total bilirubin, and CA 19-9. The surgical procedure was determined according to tumour location and extension. Either pylorus-preserving or classic pancreaticoduodenectomy was performed to resect tumours of the head or uncinate of the pancreas. Distal pancreatectomy with splenectomy was performed on lesions in the pancreatic body or tail. Total pancreatectomy was performed when the tumour extended to the head and tail. Oncology-related factors included tumour size, tumour differentiation, lymphovascular invasion, perineural invasion, stage (T, N, or M), and pathologic diagnosis.

### Statistical analysis

Statistical analyses were performed using SPSS version 21.0 (IBM, Armonk, NY). Data expressed as the mean ± standard error for continuous variables and as frequency for categorical variables. Student’s t-tests, chi-square tests or Kruskal–Wallis test were used to analyze differences between the values of continuous and categorical variables, respectively. Pearson correlation analysis was performed to evaluate the correlation between the responses of drug regimens. Pearson correlation coefficients were expressed as R. A p-value of < 0.05 was considered statistically significant.

## Supplementary information

Supplementary information.

## Abbreviations

5-FU: 5-Fluorouracil
GEM: Gemcitabine
CDDP: Cisplatinum
OXA: Oxaliplatinum
IRN: Irinotecan
DOX: Doxorubicine
PTX: Paclitaxel
DTAX: Docetaxel
EP: Epirubicin
BEL: Belotecan
TS-1: Tegafur, gimeracil
