# Supplementary material for: The identification of candidate effective combination regimens for pancreatic cancer using the histoculture drug response assay
Source: Sci Rep. 2020 Jul 20;10:12004. doi: 10.1038/s41598-020-68703-x (PMC7371642; doi:10.1038/s41598-020-68703-x)
Supplement: Supplementary file 1 — Supplementary information. [file 41598_2020_68703_MOESM1_ESM.docx]

**Figure S1.** Comparison of inhibitory rates according to buffer. (n=6, PBS; phosphate-buffered saline, DMSO; dimethyl sulfoxide.)

**Figure S2.** Comparison of inhibitory rates whether preoperative chemotherapy was administered (n=41) or not (n=5).

**Figure S3.** Comparison of IRs between combination regimens containing BEL (n=43; Kruskal-Wallis test, ** p < 0.005, *** p < 0.001).


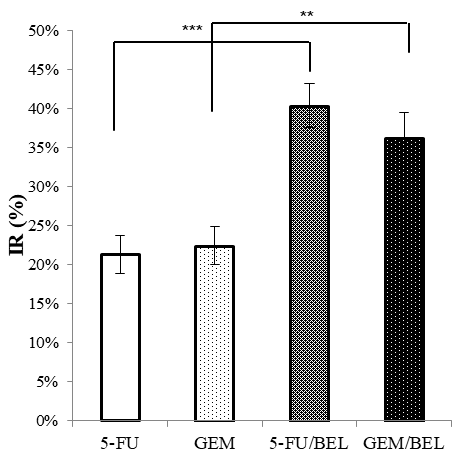


**Table S1.** Raw data generated by histoculture drug response assays (HDRAs). CDDP, cisplatinum; OXA, oxaliplatinum; IRN, irinotecan; DOX, doxorubicine; PTX, paclitaxel; DTAX, docetaxel; EP, epirubicin; BEL, belotecan; 5-FU, 5-fluorouracil; GEM, gemcitabine; TS-1, tegafur/gimeracil.

| **Raw data of single drugs and combinations regimens** | | | | | | | | | | | | |
| --- | --- | --- | --- | --- | --- | --- | --- | --- | --- | --- | --- | --- |
| Patient  number | Control | Single drugs | | Combination regimens | | | | | | | | |
|  |  |  |  | FOx | FOxIri | FBe | FBeOx | GBe | GP | GC | GD | TsOxIri |
|  | PBS | 5-FU | GEM | 5-FU OXA | 5-FU OXA IRN | 5-FU BEL | 5-FU BEL OXA | GEM BEL | GEM PTX | GEM CDDP | GEM DTAX | TS-1 OXA IRN |
| 11 | 1.002 | 0.915 | 0.773 | 0.739 | 0.631 | 0.602 | 0.568 | 0.737 | 0.451 | 0.777 | 0.830 | 0.807 |
| 12 | 0.494 | 0.390 | 0.218 | 0.220 | 0.219 | 0.342 | 0.107 | 0.161 | 0.161 | 0.170 | 0.137 | 0.195 |
| 13 | 0.247 | 0.215 | 0.206 | 0.197 | 0.197 | 0.192 | 0.151 | 0.225 | 0.194 | 0.187 | 0.206 | 0.208 |
| 14 | 0.109 | 0.081 | 0.069 | 0.079 | 0.079 | 0.055 | 0.058 | 0.075 | 0.065 | 0.062 | 0.064 | 0.065 |
| 15 | 0.509 | 0.460 | 0.476 | 0.446 | 0.428 | 0.472 | 0.372 | 0.363 | 0.328 | 0.351 | 0.472 | 0.474 |
| 16 | 0.681 | 0.584 | 0.536 | 0.563 | 0.430 | 0.569 | 0.464 | 0.567 | 0.527 | 0.386 | 0.512 | 0.375 |
| 17 | 0.945 | 0.906 | 0.902 | 0.595 | 0.555 | 0.905 | 0.794 | 0.784 | 0.898 | 0.665 | 0.664 | 0.531 |
| 18 | 0.912 | 0.745 | 0.799 | 0.722 | 0.727 | 0.654 | 0.432 | 0.608 | 0.805 | 0.533 | 0.868 | 0.469 |
| 19 | 0.982 | 0.585 | 0.632 | 0.876 | 0.540 | 0.566 | 0.466 | 0.805 | 0.738 | 0.687 | 0.897 | 0.467 |
| 20 | 0.499 | 0.424 | 0.408 | 0.295 | 0.261 | 0.370 | 0.282 | 0.406 | 0.316 | 0.268 | 0.396 | 0.211 |

**Table S2.** Drug characteristics based on the KEGG. (KEGG, Kyoto Encyclopedia of Genes and Genomes; MW, Molecular Weight.)

| **No.** | **Drug name** | | **KEGG**  **code** | **Formula** | **MW** | **Activity** | **Target** |
| --- | --- | --- | --- | --- | --- | --- | --- |
| 1 | CDDP; Cisplatinum | | D00275 | Pt(NH_3_)_2_Cl_2_ | 300.051 | Antineoplastic, Cell growth inhibitor | DNA |
| 2 | OXA; Oxaliplatinum | | D01790 | Pt.C_6_H_14_N_2_. C_2_O_4_ | 397.292 | Antineoplastic, Cell growth inhibitor | DNA |
| 3 | IRN; Irinotecan | | D08086 | C_33_H_38_N_4_O_6_ | 586.678 | Antineoplastic,  Topoisomerase I inhibitor | TOP1 |
| 4 | DOX; Doxorubicin | | D01275 | C_27_H_29_NO_11_. HCl | 579.980 | Antineoplastic,  Topoisomerase I inhibitor | TOP2 |
| 5 | PTX; Paclitaxel | | D00491 | C_47_H_51_NO_14_ | 853.906 | Antineoplastic, Tubulin depolymerization  inhibitor | TUBB |
| 6 | DTAX; Docetaxel | | D07866 | C_43_H_53_NO_14_ | 807.879 | Antineoplastic, Tubulin depolymerization  inhibitor | TUBB |
| 7 | EP; Epirubicin | | D07901 | C_27_H_29_NO_11_ | 543.519 | Antibiotic, Antineoplastic, Topoisomerase II  inhibitor | TOP2 |
| 8 | BEL; Belotecan | | D03225 | C_25_H_27_N_3_O_4_. HCl | 469.961 | Antineoplastic,  Topoisomerase I inhibitor | TOP1 |
| 9 | 5-FU; 5-Fluorouracil | | D00584 | C_4_H_3_FN_2_O_2_ | 130.077 | Antineoplastic, Antimetabolite | TYMS |
| 10 | GEM; Gemcitabine | | D01155 | C_9_H_11_F_2_N_3_O_4_. HCl | 299.659 | Antineoplastic, Antimetabolite | RRM1 |
| 11 | TS-1 | Tegafur | D01244 | C_8_H_9_FN_2_O_3_ | 200.167 | Antineoplastic, Antimetabolite | TYMS |
| 12 |  | Gimeracil | D01846 | C_5_H_4_ClNO_2_ | 145.544 | Antineoplastic, Antimetabolite | DPYD |
